# Lassa hemorrhagic fever in a late term pregnancy from northern sierra leone with a positive maternal outcome: case report

**DOI:** 10.1186/1743-422X-8-404

**Published:** 2011-08-15

**Authors:** Luis M Branco, Matt L Boisen, Kristian G Andersen, Jessica N Grove, Lina M Moses, Ivana J Muncy, Lee A Henderson, John S Schieffellin, James E Robinson, James J Bangura, Donald S Grant, Vanessa N Raabe, Mbalu Fonnie, Pardis C Sabeti, Robert F Garry

**Affiliations:** 1 Department of Microbiology and Immunology, Tulane University, New Orleans, Louisiana, USA; 2 Autoimmune Technologies, LLC, New Orleans, Louisiana, USA; 3 Corgenix Medical Corporation, Broomfield, Colorado, USA; 4 Department of Organismic and Evolutionary Biology, Center for Systems Biology, Harvard University, Cambridge, Massachusetts, USA; 5 Vybion, Inc., Ithaca, New York, USA; 6 Department of Paediatrics, Section of Infectious Disease, Tulane University, New Orleans, Louisiana, USA; 7 Ministry of Health and Sanitation Workplace Health, Republic of Sierra Leone, Freetwon, Sierra Leone; 8 The Global Viral Forecasting Initiative, San Francisco, California, USA; 9 Kenema Government Hospital Lassa Fever Ward, Kenema, Republic of Sierra Leone; 10 University of Minnesota School of Medicine, Minneapolis, Minnesota, USA; 11 Broad Institute of Massachusetts Institute of Technology and Harvard, Cambridge, Massachusetts, USA

## Abstract

Lassa fever (LF) is a devastating viral disease prevalent in West Africa. Efforts to take on this public health crisis have been hindered by lack of infrastructure and rapid field deployable diagnosis in areas where the disease is prevalent. Recent capacity building at the Kenema Government Hospital Lassa Fever Ward (KGH LFW) in Sierra Leone has lead to a major turning point in the diagnosis, treatment and study of LF. Herein we present the first comprehensive rapid diagnosis and real time characterization of an acute hemorrhagic LF case at KGH LFW. This case report focuses on a third trimester pregnant Sierra Leonean woman from the historically non-endemic Northern district of Tonkolili who survived the illness despite fetal demise.

Employed in this study were newly developed recombinant LASV Antigen Rapid Test cassettes and dipstick lateral flow immunoassays (LFI) that enabled the diagnosis of LF within twenty minutes of sample collection. Deregulation of overall homeostasis, significant hepatic and renal system involvement, and immunity profiles were extensively characterized during the course of hospitalization. Rapid diagnosis, prompt treatment with a full course of intravenous (IV) ribavirin, IV fluids management, and real time monitoring of clinical parameters resulted in a positive maternal outcome despite admission to the LFW seven days post onset of symptoms, fetal demise, and a natural still birth delivery. These studies solidify the growing rapid diagnostic, treatment, and surveillance capabilities at the KGH LF Laboratory, and the potential to significantly improve the current high mortality rate caused by LF. As a result of the growing capacity, we were also able to isolate Lassa virus (LASV) RNA from the patient and perform Sanger sequencing where we found significant genetic divergence from commonly circulating Sierra Leonean strains, showing potential for the discovery of a newly emerged LASV strain with expanded geographic distribution. Furthermore, recent emergence of LF cases in Northern Sierra Leone highlights the need for superior diagnostics to aid in the monitoring of LASV strain divergence with potentially increased geographic expansion.

## Background

LASV, a member of the *Arenaviridae *family, is the etiologic agent of LF, which is an acute and often fatal illness endemic to West Africa. There are an estimated 300,000-500,000 cases of LF each year [1-3] with a mortality rate of 15%-20% for hospitalized patients, which can become as high as 50% during epidemics [4,5] and ~90% in third trimester pregnancies for both expectant mother and fetus. Presently, there is no licensed vaccine or immunotherapy available for prevention or treatment of this disease. The severity of the disease, its ability to be transmitted by aerosol droplets, and the lack of a vaccine or therapeutic drug led to its classification as a National Institutes of Allergy and Infectious Diseases (NIAID) Category A pathogen and biosafety level-4 (BSL-4) agent. Several imported LF cases have been described since 1973, primarily from foreign nationals displaying signs of the disease upon returning to native countries or having been evacuated after falling ill abroad [6-32].

While there is no approved therapeutic for LF, the antiviral drug ribavirin has been demonstrated to reduce fatality from 55% to 5%, but only if administered within 6 days of the onset of symptoms [33,34]. The requirement for the drug to be administered at an early stage of infection to successfully alter disease outcome limits its utility given that LF has an indolent course and is difficult to diagnose by symptoms alone, particularly in the early stages where ribavirin is most effective. There is no commercially available LF diagnostic assay, which is a major challenge to early detection and rapid implementation of existing treatment regimens.

Despite the devastating effects of LF in Western African nations, to date, resources have not historically been available for the diagnosis, treatment, and monitoring of patients in country. Continuous infrastructure improvements at the KGH LFL by Tulane University, the Department of Defense (Dodd), and the United States Army Medical Research Institute of Infectious Diseases (USAMRIID) since 2005 have resulted in the implementation of sophisticated diagnostic and research capabilities at the site. Currently, the KGH LFL diagnoses LF using ELISA and LFI that detect viral antigen (Ag), and virus-specific IgM and IgG levels in the serum of every suspected case admitted to the KGH LFW. Additionally, the laboratory assesses 14 serum analyses using a Piccolo^® ^blood chemistry analyzer coupled with comprehensive metabolic panel disks. Flow cytometry powered by a 4-color Accrue^® ^C6 cytometer performs immunophenotyping, intracellular and bead-based secreted cytokine analysis. The laboratory produces its own electricity via a state-of-the-art solar collection and power generation array funded by a Coypu Foundation (New Orleans, LA, U.S.A.) grant awarded to Tulane University, and installed by South Coast Solar, L.L.C. (Metairie, LA, U.S.A.). Together, these capabilities facilitated the analysis of metabolic and inflammatory functions in real time utilizing the sera of individuals discussed in this case report with concomitant, appropriate medical intervention. Subsequently, LASV sequences amplified onsite from the serum of the afflicted LF patient were partially characterized and seemingly identified a new, significantly divergent variant of the virus from commonly circulating Sierra Leonean strains.

The case, a third trimester pregnant woman with acute hemorrhagic LF, discussed herein was closely monitored for 13 days during her hospitalization. During this period, her condition stabilized, she delivered a stillborn fetus, began walking with supervision, completed ribavirin treatment, and was awaiting discharge pending improved overall health. These studies contributed to a better understanding of the importance of and advancement in real time diagnosis and management of Lassa hemorrhagic fever in resource poor, endemic areas of Western Africa, particularly in the most highly affected subset of patients afflicted by this disease - late stage pregnant women and their fetuses.

## Methods

### Objectives

This study aimed to characterize a hospitalized acute LF case from onset of diagnosis to near full recovery using advanced rapid diagnostics and state-of-the-art technologies to dissect immune and metabolic responses in real time at the KGH LFL in Sierra Leone.

### Human Subjects

Suspected LF patients, close contacts, and healthy volunteers were eligible to participate in these studies as outlined in Tulane University's Institutional Review Board (IRB) protocol for this project, National Institutes of Health/National Institutes of Allergy and Infectious Diseases guidelines governing the use of human subject for research, and Department of Health and Human Services/National Institutes of Health/National Institute of Allergy and Infectious Diseases Challenge and Partnership Grant Numbers AI067188 and AI082119. This project was approved by the Tulane University IRB. The patients in this manuscript have given written informed consent to the publication of their case details. Patient G-1442 consented to have photographs taken at the time of admission and was informed that they may be used for illustrative purposes in scientific publications.

### Sera from suspected LF patients and healthy volunteers

Small blood volumes, typically five milliliters (mL) for serum separation and two mL uncoagulated sample were collected daily from patient G-1442, with consent from the attending physician (Donald S. Grant, M.D.), except on day nineteen. A serum sample obtained from a 20-year old pregnant woman who succumbed to LF at the KGH Maternity Ward on August 29, 2010 was used as positive control. A single sample was collected from this subject before her expiration and assigned the coded designation G-1177. Four additional sera from patients who succumbed to LF at the KGH LFW between September and December 2010 were also partially characterized (G-1209, G-1220, G-1380, and G-1401). One close contact of G-1442 was tested for Ag, IgM, and IgG and assigned the coded designation G-1446. Finally sera from healthy Sierra Leonean volunteers were used as normal controls, and assigned the coded designations LS0xx. Blood was collected in serum vacutainer tubes from patients and control donors and allowed to coagulate for 20 minutes at room temperature. Serum was separated from coagulated blood by centrifugation. The serum fraction was collected for analysis and aliquots were stored in cryovials at -20°C.

### Detection of LASV antigen by LFI diagnostic and ELISA

Serum levels of LASV nucleoprotein (NP)-specific Ag were initially measured using LASV Antigen Rapid Test cassettes and dipstick LFI currently under pre-clinical development by Corgenix Medical Corp., Broomfield, CO, U.S.A. and the Viral Hemorrhagic Fever Consortium (see acknowledgements). Both Rapid Test strip designs utilize two NP specific murine monoclonal antibodies (Autoimmune Technologies, L.L.C., New Orleans, LA, U.S.A.) in a capture and gold-conjugated detection format. An anti-murine IgG polyclonal antibody is included as a control line. The LASV Antigen Rapid Test cassettes can detect LASV NP in serum and plasma. Twenty five μL of sample were added to the sample well then chased with 100 μL of buffer. Strong titers could be detected as early as 5 minutes but final visual interpretation was conducted between 15-25 minutes of development time. The LASV Antigen Rapid Test dipsticks are similar in construction to the LFI but include a plasma separation sample pad. Whole blood from a finger stick or blood collection tubes (EDTA, citrate) was diluted 1:3 with sample buffer in a test tube followed by addition of LASV Antigen Rapid Test dipsticks. Alternatively, one drop of whole blood was added directly to the sample pad, and once the whole blood absorbed into the plasma separator material, the dipstick was placed in a test tube containing chase buffer to initiate strip development. In this format strong titers could also be detected as early as 5 minutes but final visual interpretation was conducted between 15-25 minutes of development time. Results were recorded photographically and reflectance scans were taken with a QIAGEN ESE-Quant GOLD LFI reader (QIAGEN GmbH, Hilden, Germany). Test line reflectance and Test to Control ratios (T/C Ratio) were calculated for each sample, and compared to a curve generated with recombinant quantified NP spiked into normal human serum.

The positive LF diagnosis was then confirmed with a sensitive antigen-capture ELISA employing either a murine monoclonal or caprine polyclonal capture antibody (Autoimmune Technologies, L.L.C., New Orleans, LA, U.S.A.) followed by a peroxidase-labeled caprine reagent and tetramethylbenzidine (TMB) substrate. Capture antibodies were coated in stripwell plates, blocked, dried, and packaged with desiccating packs (Corgenix Medical Corp.). A standard curve was generated with recombinant LASV NP for quantitation of serum levels of virus-associated nucleoprotein by ELISA. Sera from previously confirmed LF cases were used as positive controls. Sera from healthy Sierra Leonean and normal U.S. sera panels were used as negative controls. For analysis, sera were diluted 1:10 and incubated in wells for 60 minutes at 37°C, washed, followed by incubation with optimized HRP-labeled anti-LASV NP conjugates for an additional 30 minutes. After washing, detection was performed with TMB substrate for 15 minutes at room temperature, stopped with sulfuric acid, and read at A_450 _in a BioTek ELISA plate reader (BioTek, Winooski, VT, U.S.A.). The generation of recombinant full length LASV NP has been described elsewhere [35].

### Detection of LASV-specific serum IgM and IgG levels by ELISA

Individual recombinant LASV proteins (Vybion, Inc., Ithaca, NY, U.S.A.) and combinations optimized for detection of virus-specific IgM and IgG levels in serum were coated in stripwell plates, as outlined above. The generation of recombinant mammalian cell-expressed full length LASV GP1 and GP2 have been described elsewhere [36]. Bacterially-expressed LASV Z matrix protein was kindly provided by Dr. Erica O. Saphire, The Scripps Institute, La Jolla, CA, U.S.A. Sera from suspect and convalescent LF cases previously characterized for LASV antigen-specific IgM and IgG responses were used as positive controls in respective ELISA formats. Sera from healthy Sierra Leonean volunteers without significant titers against LASV antigens, and normal U.S. sera panels were used as negative controls. For analysis, sera were diluted 1:100 and incubated in wells for 30 minutes at room temperature, washed, followed by incubation with optimized HRP-labeled anti-human IgG or IgM conjugates for an additional 30 minutes. After washing, detection was performed with TMB substrate for 10 minutes at room temperature, and read as described above.

### Comprehensive Metabolic Panel analysis

The kinetics of fourteen serum analyses were analyzed daily using a Piccolo^® ^blood chemistry analyzer (Abaxis, Inc., Union City, CA, U.S.A.) with Comprehensive Metabolic Reagent Discs, as per manufacturer's recommendations.

### Cytokine kinetics

Kinetics of eleven serum cytokines were analyzed with an Accrue C6^® ^benchtop cytometer (Accrue Cytometers Inc., Ann Harbor, MI, U.S.A.) and an eBioscience FlowCytomix Human Th1/Th2 11-plex Kit (Bender MedSystems GmbH, Vienna, Austria). Serum aliquots collected and frozen throughout the timeline were analyzed concurrently at the end of the study.

### Urinalysis

Ten separate urinalysis tests were performed daily within 20 minutes of urine collection, except for the last two days of this study timeline, using a VWR^® ^Urine Reagent Strips (VWR, Arlington Heights, IL, U.S.A.).

### qPCR

RNA was extracted from serum using QIAmp Viral RNA Mini kit (QIAGEN, Valencia, CA, U.S.A.). RT-PCR was performed using SuperScript III (Invitrogen, Carlsbad, CA, U.S.A.) and qPCR was performed with PerfeCTa SYBR Green (Quanta Biosciences, Gaithersburg, MD, U.S.A.) using primers 36E2 and 80F2 directed against the LASV GPC gene [37]. A seed stock of Josiah LASV strain (kindly provided by Dr. Lisa E. Hensley, Viral Therapeutics Branch, Virology Division, USAMRIID Diagnostic Systems Division, Fort Detrick, MD, U.S.A.) was used as a standard for calculating RNA copies of LASV present in the serum samples.

### Sequencing and phylogenetic analyses

The entire LASV S segment was amplified using primer CGCACAGTGGATCCTAGGCAT. Standard Sanger sequencing was then performed using primer G2 targeting the glycoprotein complex (GPC) gene [38]. Alignments from patient G-1442 and 73 partial GPC sequences were created using Muscle [39] followed by manual adjustments. A Neighbor-joining tree was created using LASV Pinneo as an outgroup, and bootstrapped over 1000 replicates.

### Statistical methods

ELISA data were plotted in MS Excel as mean ± SD, N = 2, with error bars. Analysis between time points was performed with Analysis of Variance (ANOVA). Cytokine levels were calculated by curve fitting analysis of data generated with quantified standards for each analyte.

## Results

### Case presentation

**Figure 1:**
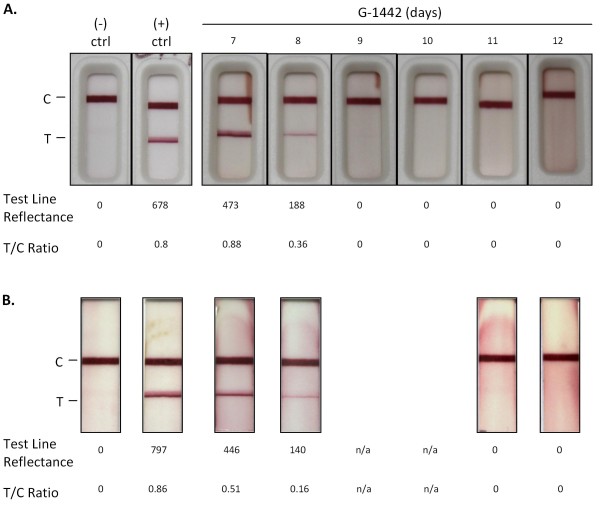
**Rapid diagnosis of acute LF virus infection by LFI in patient G-1442**. LFI diagnostic (A) and dipstick (B) tests detected LASV NP in the serum of suspected LF patients. After 15 minutes of development the results were recorded photographically and reflectance scans were taken as outlined in Methods. A representative normal serum sample analysis from a Sierra Leonean donor (- ctrl) is shown for comparison. Only the control line developed with this serum sample. Conversely, sera from G-1442 generated a detectable precipitate in the test line, indicative of LASV NP antigen. The positive control (+ ctrl) was recombinant LASV NP diluted in sample buffer. The LFI diagnostic and dipstick platforms detected NP antigen on days 7-8. Days 9-12 show no detectable antigen in either format. Test line reflectance and Test to Control ratios (T/C Ratio) are indicated below each test.

On January 20^th^, 2011 the KGH Maternity Ward alerted the LF team of a suspected case from Tongo, lower Bambara chiefdom in Kenema district, Sierra Leone. A blood sample was collected and sent to the KGH LFL for testing. LASV NP antigen LFI diagnostic confirmed LF within 20 minutes of sample processing (Figure 1). The patient was a 22-year old pregnant woman, estimated gestational age 32 weeks, who had recently travelled to Tongo from Mabineh 1 village, Kunike chiefdom, Tonkolili district, northern Sierra Leone (Additional File 1, Figure 1). She arrived in Tongo on January 10^th ^experiencing fever and lower abdominal pain. She was taken to Tongo Maternal Health Post on January 15^th ^for observation where she was referred to KGH on January 19^th ^as a maternity case after failing to respond to treatment with antibiotics (Ampicillin and Gentamycin). The KGH Maternity Ward staff suspected LF upon arrival and referred the case to the LFW and LFL. This patient was assigned the coded designation G-1442, which will be used henceforth.

Case G-1442 presented with symptoms of fever, sore throat, headache, red eyes, weakness, facial edema, retrosternal pain, generalized abdominal pain, epistaxis and haemoptysis (Additional File 2, Figure 2). On examination, her body temperature was 36.5°C, pulse rate of 96 beats/minute, respiration rate of 26/min, and blood pressure of 90/40 mm Hg (Additional File 3, Figure 3). Respiratory findings included nasal flaring and bibasal crepitations. Abdominal findings included a hard uterus that was tender to palpation with an estimated symphysis fundal height of 30-32 weeks. There was marked epigastric tenderness. Minute bilateral conjunctival hemorrhages were also noted. The differential diagnosis included probable LF, pneumonia, and a possible concealed antepartum hemorrhage (concealed placental abruption).

**Figure 2:**
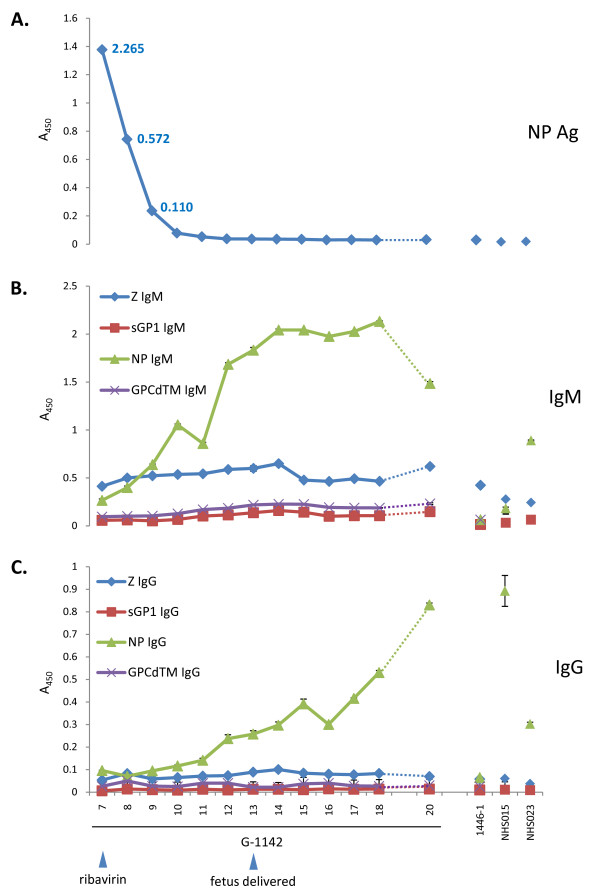
**Nucleoprotein, virus-specific IgM and IgG detection by ELISA in G-1442, normal, and contact G-1446 sera**. An Ag capture ELISA was used to detect LASV NP in patient sera (A). LASV NP Ag was not detected in normal sera from Sierra Leonean origin, or in contact G-1446. The level of LASV NP Ag [blue diamond] in G-1442 dropped significantly during the first 3 days of ribavirin administration, and was undetectable by day 10. LASV-specific IgM (B) and IgG (C) were assayed in a recombinant ELISA plate format, with individually coated NP, GP1 (sGP1), GP2 (GPCΔTM), or Z proteins. One Sierra Leonean serum registered a high IgG titer to NP (NHS015), whereas the other had moderate IgM titers to NP (NHS023), but both were negative for IgG and IgM to Z and glycoproteins. NP-specific IgM and IgG levels in G-1442 rose throughout the course of the illness, through day 20. Contact G-1446 did not have measurable IgG titers, and only registered a low IgM titer to Z. Data are plotted as mean A_450 _± SD, N = -2. The line between day 18 and 20 is dotted to reflect discontinuity on day 19.

**Figure 3:**
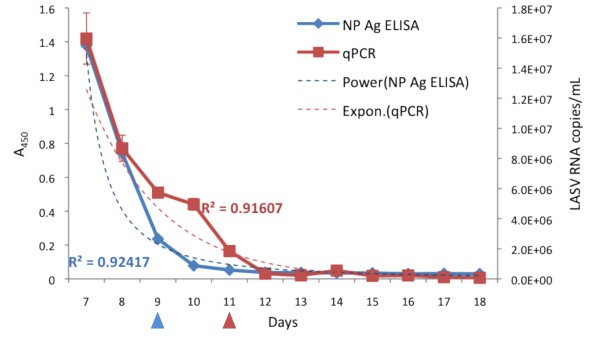
**Comparison of LASV NP antigen detection by ELISA versus RNA quantification by qPCR**. RNA was prepared from serum samples as outlined in materials and methods. RT-PCR followed by qPCR directed against the GPC gene was performed on days 7-18. A 1:6 dilution series of Josiah strain seed stock was used as a standard to calculate the LASV RNA copy number per milliliter of serum. PCR data were plotted on the second Y axis (LASV RNA copies/mL). Error-bars represent the SEM of two independent experiments. NP Ag ELISA data was plotted on the first Y axis (A_450_) for trend comparison. Trend lines for NP Ag ELISA (power) and qPCR (exponential), and associated R^2 ^values are indicated. The limit of detection for antigen by NP Ag ELISA was day 9 (blue arrow), and day 11 for qPCR (red arrow).

### Travel history and contact tracing

The case patient had travelled from Mabineh 1 to Waterloo (south of Freetown) three weeks prior to her illness. Upon returning from Waterloo she resided in Massingbi (a neighboring town to Mabineh 1) for one week before returning to Mabineh 1. She remained in Mabineh 1 for five days before departing for Tongo. According to relatives it is estimated that the case patient left Mabineh 1 between January 6^th ^and 8^th ^bound for Tongo (Additional File 1, Figure 1C). She had no known exposure to an ex-LF patient or contact with rodents prior to her illness. However, an assessment of her previous dwelling in Mabineh 1 revealed evidence of rodent waste, and rat holes in a structure constructed with mud and with large open spaces in walls. The patient had not been seen by a medical professional throughout her pregnancy as nurses at the Mabineh Health Post could not account for her visiting the center at anytime over the previous eight months. The date of onset of LF was recorded as January 13^th^, with fever, headache, and lower abdominal pain, after failure to respond to treatment with anti-malarials. The patient tested positive for malaria parasites while in Tongo (verbal communication). The conclusion from the investigation conducted by the LF outreach team points to infection with LASV in the northern towns of Massingbi and Mabineh 1 where the case patient resided during most of the early stage of the incubation period of the disease.

Contacts of the case patient were identified and none have developed symptoms of LF to date. All contacts were monitored throughout the incubation period (21 days) from date of last reported exposure. Contacts were family members from Mabineh 1, nursing staff at the health clinic in Tongo, and the patient's brother who resides in Tongo township. The brother of G-1442, designated G-1446, with whom she resided while in Tongo, accompanied her to the KGH and was tested for LASV antigen, IgM, and IgG. He tested negative for all three (Figure 2). Testing of G-1446 was prompted by his close contact with G-1442 in Tongo for 9 days, and given the hemorrhagic presentation, with vomiting by the latter at the time of admission to KGH. Additionally, G-1442's mother traveled from Mabineh 1 to Kenema to assist with her daughter's care during hospitalization at the KGH LFW. The mother did not develop a fever and did not feel ill at any time over the course of nearly two weeks of permanence in Kenema; therefore, she was not tested for LASV antigen or immunoglobulin levels. The patient revealed that she travelled from Masingbi to Tongo by motorcycle over the course of 2 days. The motorcycle operator was an unidentified male, and further information on his whereabouts and health status is not known.

The geographical location where G-1442 contracted LF is of particular importance. In recent months several cases have been identified by our field research team in the northern districts of Bombali and Tonkolili (Additional File 1, Figure 1A), which have not been previously considered endemic regions for the illness. Since the Fall of 2010, however, two cases of severe hemorrhagic LF have been identified in these two northern districts, both with fatal outcomes. In addition, several other LF cases from the same districts have been confirmed with subsequent treatment at the KGH LFW and positive outcomes. During the preparation of this manuscript, additional LF cases had been diagnosed at the Magbeneth Hospital in Makeni using LASV Ag Rapid LFI diagnostics provided by Tulane University and Corgenix Medical Corporation.

### Diagnostic Analysis

A blood specimen collected on patient G-1442's day of admission was positive for LASV NP Ag by LFI diagnostic (Figure 1), and by quantitative NP capture ELISA, with a level of 2.265 μg/mL NP (Figure 2). The LFI platform confirmed acute LF within 20 minutes of sample collection. IgM levels to recombinant LASV proteins (NP, GP1, GP2, Z) were determined by ELISA, with low but detectable levels of immunoglobulin to NP and Z (Figure 2B). This data suggests the patient was naive to LASV exposure prior to this incident. Statistically significant levels of low IgM response to GP1 and GP2 were detected on days 11-20 when compared to naïve negative controls and G-1142 sera from days 7 - 10 (p < 0.05) (Figure 2B). Low levels of NP-specific IgG were not detected until at least day 12 post onset of illness (Figure 2C). During the monitoring period G-1442 did not develop significant IgG titers against GP1, GP2, and Z.

LASV NP antigen dropped rapidly over 3 days following treatment with ribavirin, and was below the limit of detection (LOD) of the assay by day 10 in an NP Ag capture ELISA (Figure 2A). RNA was isolated from serum on the day of collection and analyzed by qPCR for amplification of a conserved 300 nt segment of the LASV GPC gene. PCR confirmed and detected viral RNA in serum samples at least 2 days beyond the NP Ag ELISA (Figure 3). Overall, the NP Ag capture ELISA, LFI diagnostic, and qPCR assay showed the same trend with decreasing titers of LASV following the start of ribavirin treatment.

### Clinical Chemistry

**Figure 4:**
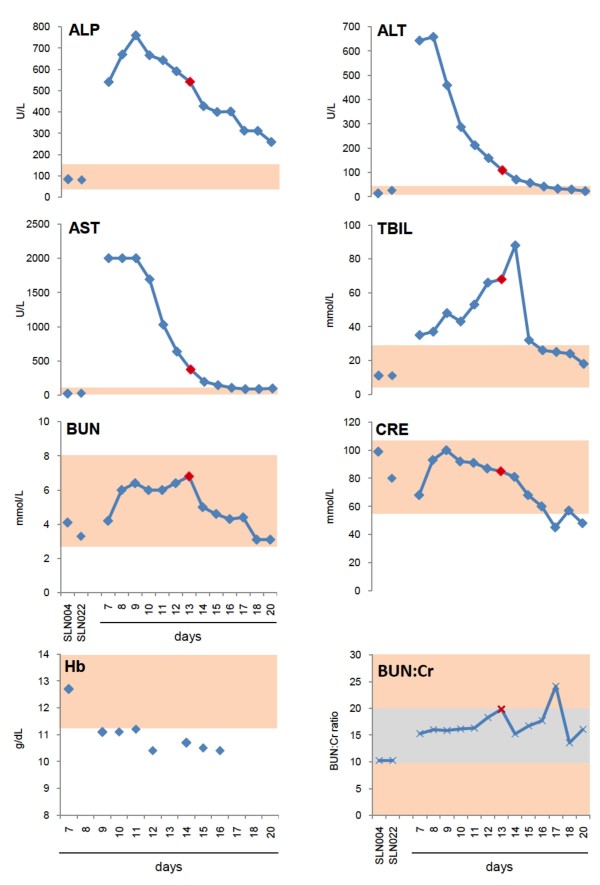
**Comprehensive daily Piccolo metabolic panel analysis**. Metabolic indicators were measured in the serum of G-1442 daily after admission, through day 20 (with the exception of day 19), using a Piccolo comprehensive metabolic panel disk array. Two Sierra Leonean normal controls were also analyzed for comparison (SLN004 and SLN022). Values were plotted alongside normal ranges for each metabolite (rose boxes), for reference. G-1442 presented with normal BUN, CRE, but elevated TBIL. Analytes ALP, ALT, and AST were highly elevated upon admission, all indicative of severe liver implication in this LF case. Metabolic indicators in the two healthy Sierra Leonean donors were within normal ranges. Hemoglobin levels were independently measured in G-1442 on days 7, 9-12, and 14-16 post-onset of disease. The day of stillbirth delivery is indicated in each panel by a dark red diamond (day 13).

On presentation to KGH LFW, the liver function panel revealed highly elevated levels of aspartate aminotransferase (AST) >2000 U/L, alanine transaminase (ALT) of 643 U/L, alkaline phosphatase (ALP) of 541 U/L, and total bilirubin (TBIL) of 35 micromoles per liter (μM/L) (2.05 mg/dL) (Figure 4). Levels of sodium, potassium, chloride, calcium, carbon dioxide (TCO_2_), blood urea nitrogen (BUN), and total protein were within or near normal levels, and albumin was below normal range (Additional File 4, Figure 4). At presentation the hemoglobin level (Hb) was 12.7 g/dL (Figure 4).

### Treatment and hospital course

Intravenous ribavirin was administered upon NP Ag positive diagnosis by LFI: a loading dose of 30 mg/kg followed by 15 mg/kg every six hours for four days, followed by 7.5 mg/kg every eight hours for six days. Amoxicillin, intravenous quinine in 5% dextrose, acetaminophen, and routine vitamins (multivitamin, ferrous sulphate, folic acid) were commenced upon admission to KGH. Patient G-1442 developed bleeding from the oral mucosa on day eight which resolved on day ten. On day ten she was much improved and was able to stand unaided. Fetal demise was confirmed with the aid of a fetal heart Doppler on day twelve. She had an uncomplicated vaginal delivery of a stillborn fetus on day thirteen, at which time she was started on ampicillin and metronidazole. Artesunate was begun on day fifteen. IV fluid boluses of five percent and 50% dextrose and Ringer's Lactate solution were given as needed.

The sodium and chloride levels gradually decreased during the hospitalization period, following IV fluids management, and G-1442 remained hyponatremic between days 13 and 20 and hypochloremic between days 16 and 20. Creatinine and BUN remained normal during her entire hospital stay resulting in a BUN:Cr ratio between 10 and 20 except for a transient increase to 24 on day 17 (Figure 4). The ALT declined steadily during hospitalization, and was within normal levels on day 16 (42 U/L) and through the rest of the monitoring period, while the AST remained >2000 U/L for three days before declining to 100 U/L on day 20. The ALP remained elevated as well, decreasing to 259 U/L on day 20. The TBIL initially increased to 88 μM/L (5.15 mg/dL), before dramatically declining two days after delivery (day 14) to 32 micromoles per liter (1.87 mg/dL). Bilirubin continued to decrease over the remainder of the observation period, to within normal levels between days 16 and 20. The Hb decreased from an initial normal level of 12.7 g/dL to between 10.4 and 11.1 g/dL throughout the hospitalization period (Figure 4B). These Hb levels did not prompt the doctor to administer a blood transfusion.

**Figure 5:**
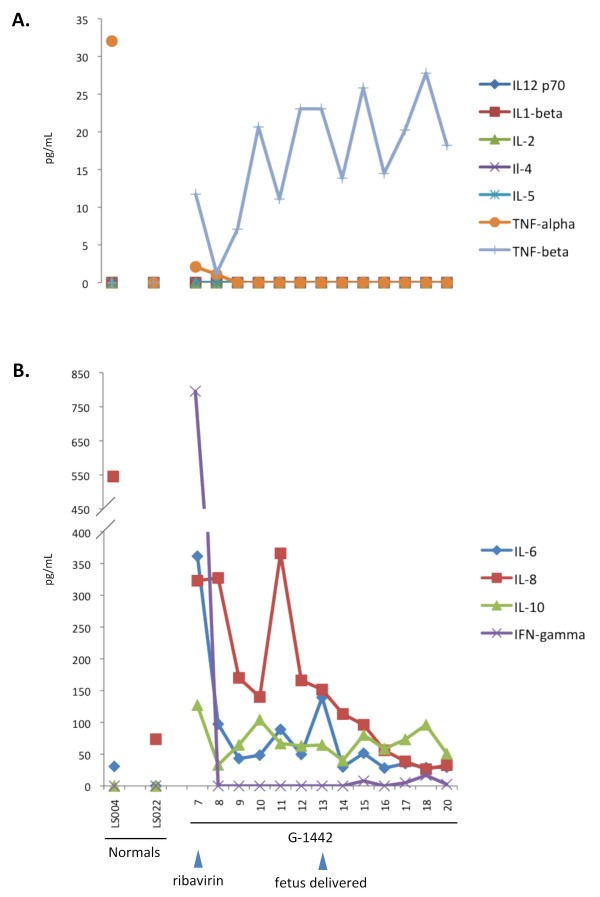
**Serum cytokine levels analyzed by multiplex Flow Cytometry**. Data generated with a Human 11-Plex Inflammatory Cytokine kit was quantified with Flow Cytomix Pro software, and plotted on linear scales. G-1442 presented with elevated levels of TNF-β(A.), IL-6, IL-8, IL-10, and notably IFN-γ(B.), all of which decreased by the next day, following initial treatment with ribavirin (day 7, arrow). Cytokine levels were also measured in normal Sierra Leonean controls, for comparison (LS004, LS022). Significant changes in the cytokine profile were not noted following delivery of a stillbirth fetus (day 13, arrow).

Cytokine profiles were performed on serum samples collected daily (Figure 5). G-1442's IFN-γ levels were highly elevated on the day of admission, but decreased to baseline levels by the following day and did not rise above normal levels over the ensuing 12 days of monitoring (Figure 5B). A significant decrease in IL-6 and IL-10 levels was noted on day 8, but levels fluctuated throughout the course of hospitalization. IL-8 levels dropped significantly on days 9 and 10, followed by a spike on day 11, and a steady decrease thereafter. TNF-β was present at elevated levels at the time of admission and decreased to near background levels by the following day, but increased significantly and steadily throughout the hospitalization period. Interleukins -1β, -2, -4, -5, 12p70, and TNF-α were not detected or were present in the serum of G-1442 at very low levels at all time points analyzed. On day 20, the last day of monitoring, all cytokines with the exception of TNF-β were at or near normal levels. Interleukin-8 and TNF-α were elevated in one healthy control serum (LS004) but were within normal levels in the other healthy control serum (LS022). All other cytokines were at baseline levels in both control sera (Figure 5A, B).

### Urinalysis profile

Urinalysis revealed ongoing proteinuria (30 mg/dL) that peaked on the day of delivery (300 mg/dL) and subsequently decreased (trace to 30 mg/dL). It is unclear whether proteinuria resulted from LASV infection, pregnancy, or a combination of factors. There was no hypertension to suggest a diagnosis of pre-eclampsia. The presence of large blood was noted on all urinalysis results. Microscopy was not performed to examine for red blood cells or casts. Bilirubin (small to large) was present prior to and including the day of delivery, after which time it was absent, consistent with resolving biliary obstruction as indicated by the decrease in serum TBIL levels and ALP. Leukocyte esterase was absent to trace presence prior to delivery, after which time small to moderate results were noted. Since analysis was performed on catch specimens, the possibility of contamination from vaginal fluid after delivery cannot be excluded. Nitrites were positive on day 18. No symptoms of urinary tract infection were noted (Additional File 5, Table 1).

### Sequencing analysis and strain characterization

**Figure 6:**
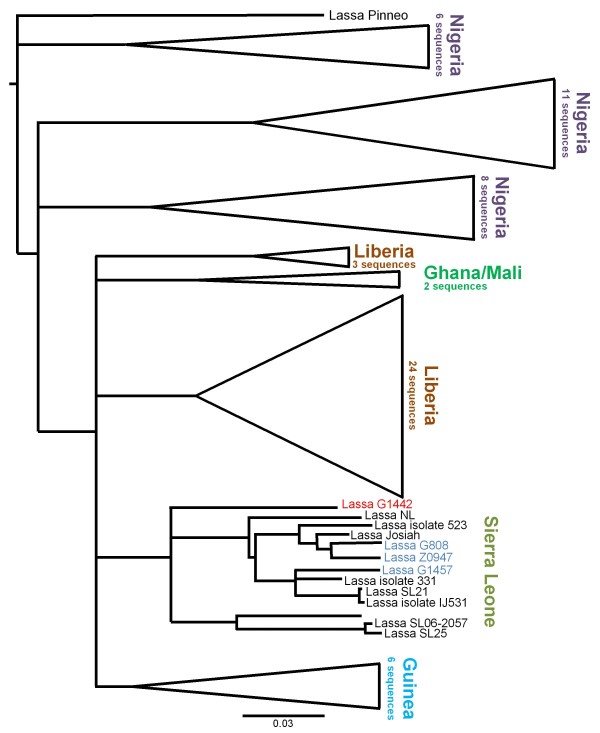
**Phylogenetic analysis of LASV from patient G-1442**. A ca. 800 bp fragment of the GPC gene was sequenced and aligned to 73 other sequences available in the NCBI database. A Neighbor-joining tree using LASV Pinneo as an outgroup was created with 1,000 replicates of bootstrap and clades from different countries are displayed as cartoons. Recent LASV isolates from the Kenema area are marked in blue. The scale bar indicates 3% nucleotide divergence.

In order to get a better idea of the geographical location of the isolated strain, a 800nt fragment of the GPC gene from the serum of patient G-1442 was sequenced and compared to other strains circulating in Sierra Leone and West Africa. Phylogenetic analysis showed that the LASV from patient G-1442 clustered with other strains from Sierra Leone, but was significantly different from all of them, forming its own sub-group (Figure 6). The strain was only 89% identical to the prototypical Sierra Leonean LASV Josiah strain at the nucleotide level. In comparison, we have found other currently circulating strains from Sierra Leone to be more than 95% identical to Josiah (Figure 6 and data not shown). This suggest that a new strain of LASV may be responsible for the recent outbreak of LF in the North of Sierra Leone, although more complete sequencing studies are required to firmly establish this.

### Chemistries of healthy volunteers and five fatal cases of LF

In order to establish the capabilities and reliability of the Piccolo^®^, complete chemistries were performed on blood drawn from two healthy Sierra Leonean volunteers as well as on samples from five patients who succumbed to LF (Additional File 6, Table 2). Patient G-1177's metabolic and biochemical characterization was described in detail elsewhere [40]. The chemistries of the two healthy volunteers were in the normal range as specified by the manufacturer (Abaxis, Inc.) (Additional File 6 Table 2).

LF patients G-1209, G-1220, G-1380, G-1401, and G-1177 had extremely abnormal labs prior to expiration (Additional File 6, Table 2). Along with dysregulated serum electrolytes, all subjects had a highly elevated liver panel, and, with the exception G-1380, all had elevated levels of BUN. Additionally, all subjects had low serum albumin and total protein levels. The cytokine profiles between healthy volunteers and subjects who succumbed to LF were largely unremarkable, with the single exception of IL-10, which was recorded at elevated levels in all cases (Additional File 6, Table 2 and unpublished data).

## Discussion

LASV Ag Rapid Test detected acute LASV infection in G-1442 within 20 minutes of serum collection and processing at the KGH LFL (Figure 1). The patient was immediately transferred from the KGH Maternity Ward to the LFW upon diagnosis, permitting isolation and appropriate medical intervention including IV ribavirin administration, currently the only drug used in viremic cases of LF. LFI diagnostic detected LASV NP Ag on the first two days at the KGH LFW (Figure 1), whereas antigen capture ELISA diagnostic detected the protein in the serum of G-1442 for three days following admission (Figure 2A). Quantitative PCR extended detection of LASV RNA sequences for two days beyond the limit of detection of LASV NP Ag ELISA, thus establishing a role for each platform from sensitive and rapid point of care LFI diagnostic to ultrasensitive and time extended qPCR detection of very low levels of arenaviral RNA in the blood.

ELISA data suggest that patient G-1442 was naïve to infection as she presented with very low LASV-specific IgM to all viral proteins analyzed at 7 days after onset of symptoms; she then began showing a consistent increase in NP-specific IgM, and a low level IgM response against the glycoproteins starting on day 11, which continued through all days monitored. Only IgG to NP developed over the analysis timeline (Figure 2C). The predominant, mature, humoral response in LF is against the viral NP Ag [41-43, unpublished data].

The metabolic panel of G-1442 as well as a previously characterized severe hemorrhagic LF case, G-1180, who also survived [40], show important differences with patients who succumb to the disease. Despite hepatic and renal dysfunction during the course of LF infection, neither patient developed elevated levels of serum CRE, which are usually associated with a poor outcome [18]. In G-1442 the BUN:Cr ratio remained within normal levels throughout (10-20:1), with the notable exception of day 17, when it rose above 20 (24.2). These data suggest that in G-1442 renal function was not significantly affected by LF. Conversely, G-1177, a late term pregnant woman diagnosed with LF in August 2010, succumbed to the disease with a CRE level of 818 μmol/L and a BUN:Cr of 5.6 prior to expiring, which is indicative of significant intrarenal damage Additional File 6, Table 2]. Another significant discrepancy between the two pregnant LF cases was the measured levels of AST. In G-1177 the single sample AST level was zero, whereas G-1442 had a highly elevated level of AST at the time of admission (>2,000 U/L), which rapidly resolved over the course of treatment (Figure 4). Levels of AST are commonly highly elevated in LF cases, thus the undetectable level in G-1177 may have been indicative of severe liver failure near the time of expiry and not a representative hepatic metabolic state in late term pregnancies afflicted by LASV infection. Both surviving patients, G-1442 and G-1180, showed rapid resolution of severe hepatic dysregulation, measured by ALP, ALT, and AST, to within normal or near normal levels at the conclusion of ribavirin treatment.

At the time of admission G-1442 presented with elevated serum levels of IFN-γ, IL-6, IL-8, and TNF-β (Figure 5A, B). Elevated IFN-γ and IL-6 levels are common in non-lethal LF and other febrile illnesses alike, but are highly variable in fatal cases of LF [18,44]. Elevated IL-8 levels have been associated with positive outcomes in acute LF, but are also common in native Sierra Leonean healthy controls [44, unpublished data]. Spontaneous cytokine production in acutely ill and healthy persons living in endemic areas for Human Immunodeficiency Virus, Malaria, Yellow Fever, Dengue, and assorted parasitic infections, has been reported [45], thus prompting evaluation of such immunomodulatory molecules in the context of specific disease states. Measurable and sustainable levels of TNF-β in G-1442 are a distinguishing feature among the LF cases characterized to date. Detection of TNF-β in G-1442 but not in any of the approximately 100 additional LF patients analyzed in our studies thus far (unpublished data) may represent a rare immunological response to the febrile illness, may be associated with the pregnant status of this patient, may have manifested because of a response to a co-infecting pathogen, or may be a combination of factors. The anti-inflammatory cytokine IL-10 was elevated in G-1442's serum throughout the treatment period. Interleukin-10, a stimulator of B cell maturation and antibody production, is commonly recorded in LF patients when IgM and IgG responses to LASV antigens emerge [18,44], irrespective of outcome. Interleukin-1β was not detected in G-1442 throughout the course of recovery from LF. This observation generally contrasts with previous LF studies showing that IL-1β was significantly elevated in non-fatal versus fatal LF and non-LF febrile illness, but not in healthy controls [44].

Patient G-1442's test results, in conjunction with those obtained for G-1180 [40], strengthen the hypothesis, as previously proposed by others, that an imbalance between pro- and pre-inflammatory cytokines plays an important role in the development of Lassa hemorrhagic shock, with poor outcome [18,44]. As observed with G-1180, the marked absence of TNF-α, a potent inducer of endothelial damage via apoptosis [46] and thrombocytopenia [47], throughout the monitored course of G-1442's illness, suggests a regulated and effective immune response at play. These studies also suggest that lack of specific physiological responses, e.g. elevated TNF-α, serum CRE, and BUN levels, may be relevant, early predictors of outcome in hemorrhagic LF. It is also noteworthy that G-1442 did not present with high core temperature, which remained at or below 36.5°C throughout the acute phase of the illness despite a febrile diagnosis (Additional File 3, Figure 3) and high IFN-γ levels (Figure 5B). Her body temperature then fluctuated between 36°C and 37.5°C from day 15 onward.

Together, these data strengthen the potential for increased positive outcomes in cases of severe hemorrhagic LF. More importantly, it outlines the possibility of adequate disease management with positive outcome in third trimester pregnancies, particularly for the mother [48]. Despite severe and prolonged multi-organ dysregulation, pro- and anti-inflammatory cytokine up- and down-regulation, management of a 32 week-pregnancy, a stillbirth delivery, and overall poor health, patient G-1442 was recovering well on day 20 and was discharged on day 25. A quick diagnosis of acute LF followed by prompt treatment with IV ribavirin, IV fluids management, maintenance of electrolyte balance to counter hypovolemia, hemorrhagic shock, malnutrition, and adequate control of secondary infections, even 7 days post onset of symptoms in a severe case of the illness, can meet with a positive outcome.

Additionally, this study highlights the emergence of LF cases in the northern districts of Sierra Leone, where the disease has not been widely reported or identified. Recent collaborative efforts with staff at the Magbeneth Hospital in Makeni includes beta-testing of LASV Ag Rapid Test LFI diagnostic modules, community sensitization, and prompt reporting of antigen positive LF diagnoses to the KGH LFW for patient transport, isolation, and treatment and may be a contributing factor to the elevated number of reported cases in northern Sierra Leonean districts. With promising new diagnostics, we are able to both enhance care of patients in the clinical setting and increase our understanding of the range and impact of this devastating disease. The continuous capacity building at the KGH LFL also permits real time analysis of viral RNA levels by qPCR, cDNA generation, followed by high-throughput next-generation sequencing. Although more extensive studies will be required before confirming the emergence of new LASV strains, particularly in the historically non-endemic northern districts of Sierra Leone, sequencing efforts in this case point to divergence of circulating strains throughout the country, with possible widening in geographical distribution.

## Competing interests

The authors declare that they have no competing interests.

## Authors' contributions

Conceived and designed the experiments: LMB, MLB, KGA, RFG. Performed the experiments: LMB, MLB, KGA. Analyzed the data/critical review of manuscript: LMB, MLB, KGA, JNG, JSS, JER, DSG, VNR, PCS, RFG. Contributed reagents/materials: IJM, LAH. Provided medical/outreach/case investigation support in Sierra Leone: LMM, JJB, DSG, VNR, MF. Wrote the manuscript: LMB, MLB, KGA, JNG, RFG. All authors have read and approved the final manuscript.

## Supplementary Material

Additional File 1 **Map of Sierra Leone and expanded view of relevant localities and routes travelled by patient G-1442**. Maps of Sierra Leone outlining Districts (A) and Provinces (B) [http://commons.wikimedia.org/wiki/Atlas_of_Sierra_Leone], with an inset map (C) [http://maps.google.com] displaying the location of Mabineh 1 [red star], where the suspected LF case in the current report originated, and the four localities where the patient travelled to and from, with known dates noted: Waterloo (late Dec 2010), Masingbi (early Jan 2011), Tongo (Jan 10, 2011), and Kenema (Jan 19, 2011). The inbound routes travelled by the patient are indicated in dotted lines, and outbound ones in solid lines. The bar represents 20 miles. Click here for file

Additional File 2 **Patient G-1442 at time of admission presenting with haemoptysis, facial edema, gingivorrhagia**. Patient G-1442 presented with significant haemoptysis, facial edema, and gingivorrhagia, at the time of admission and medical assessment at the KGH LFW. These symptoms persisted for several days after admission but resolved with ribavirin treatment. Click here for file

Additional Figure 3 **Vital signs for G-1442 during hospitalization at KGH LFW**. Core temperature (°C) [green triangle], pulse [red circle], respiratory rate [blue diamond], and blood pressure purple [square = systolic, yellow square = diastolic] were measured at regular intervals, usually every 4 hours at the onset, and every 12 hours at later times, throughout the hospitalization period. Click here for file

Additional Figure 4 **Additional Piccolo metabolites analyzed in G-1442**. Patient G-1442 presented with low serum Cl^- ^and albumin, normal K^+^, Na^+^, Ca^2+ ^(corrected for albumin levels), TCO_2_, and total protein levels. Over the course of disease management the patient developed hyponatremia, hypochloremia, and slight hypokalemia. Total protein and albumin levels remained low throughout. Between days 9 and 13 G-1442 developed hypercalcaemia, but then normalized. Metabolic indicators in the two healthy Sierra Leonean donors all were within or near normal ranges. Click here for file

Additional Figure 5 **Table 1. Urinalysis profile for patient G-1442 during the course of admission at the KGH LFW**. Urine samples were collected from patient G-1442 daily (days 7-18) and tested for 10 metabolites as outlined in Methods. The first day of ribavirin administration (7) and still birth delivery (13) are noted. Abbreviations and codes: moderate (mod.); negative (-); positive (+); specific gravity (spec. gravity); 30 mg/dL protein in urine (30+); 300 mg/dL protein in urine (300+). Click here for file

Additional Figure 6 **Table 2. Metabolic, cytokine, LASV Ag, IgG, and IgM profiles for five patients who succumbed to LF at the KGH LFW in recent months, and in two healthy controls**. Thirteen metabolic indicators, 11 cytokines, LASV NP Ag, IgM, and IgG status were compared between 5 representative recent fatal cases of LF (G-1209, G-1220, G-1380, G-1401), including one previously characterized fatal late term pregnancy (G-1177), and two healthy volunteers (LS004, LS022). Reported normal ranges for metabolic indicators (Abaxis, Inc.) and serum cytokine levels (Cambridge Biomedical [IL-1b, IL-10], BD Biosciences [IL-2, IL-4, IL-8, IL-12p70], R&D Systems [IL-5, IL-6], Thermo Scientific [TNF-α], BioVendor [TNF-β, IFN-γ]) are shown in the rightmost corresponding columns. Metabolic panel values are in SI units, and cytokine levels are in pg/mL. ELISA data was scored as positive (+), negative (-), or indeterminate (+/-), based on statistical comparison to positive and negative sera, and using a positive control serum dilution series. Click here for file

## References

[B1] Buckley SM Casals J Lassa fever, a new virus disease of man from West Africa. Isolation and characterization of the virus Am J Trop Med Hyg 1970 19 4 680 691 4987547 10.4269/ajtmh.1970.19.680

[B2] Birmingham K Kenyon G Lassa fever is unheralded problem in West Africa Nat Med 2001 7 8 878 11479607 10.1038/90892

[B3] Fisher-Hoch SP McCormick JB Lassa fever vaccine: A review Expert Rev Vaccines 2004 3 103 111 10.1586/14760584.3.2.189 15056044

[B4] McCormick JB King IJ Webb PA Johnson KM O'Sullivan R Smith ES Trippel S Tong TC Sacchi N A case-control study of the clinical diagnosis and course of Lassa fever J Infect Dis 1987 155 3 445 455 10.1093/infdis/155.3.445 3805772

[B5] McCormick JB Epidemiology and control of Lassa fever Current Topics in Microbiol and Immunol 1987 134 69 78 10.1007/978-3-642-71726-0_3 3581899

[B6] Haas WH Breuer T Pfaff G Schmitz H Kohler P Asper M Emmerich P Drosten C Golnitz U Fleischer K Gunther S Imported Lassa fever in Germany: surveillance and management of contact persons Clin Infect Dis 2003 10 1254 1258 10.1086/374853 12746770

[B7] Holmes GP McCormick JB Chase RA Lewis SM Mason CA Hall PA Brammer LS Perez-Oronoz GI McDonnell MK Lassa fever in the United States. Investigation of a case and new guidelines for management N Engl J Med 1990 323 16 1120 1123 10.1056/NEJM199010183231607 2215580

[B8] Amorosa V Macneil A McConnell R Patel A Dillon KE Hamilton K Erickson BR Campbell S Knust B Cannon D Miller D Manning C Rollin PE Nichol ST Imported Lassa Fever, Pennsylvania, USA, 2010 Emerg Infect Dis 2010 16 10 1598 1600 20875288 10.3201/eid1610.100774 PMC3294406

[B9] Atkin S Anaraki S Gothard P Walsh A Brown D Gopal R Hand J Morgan D The first case of Lassa fever imported from Mali to the United Kingdom, February 2009 Euro Surveill 2009 14 10 12 19317988

[B10] Kitching A Addiman S Cathcart S Bischop L Krahé D Nicholas M Coakley J Lloyd G Brooks T Morgan D Turbitt D A fatal case of Lassa fever in London, January 2009 Euro Surveill 2009 14 6 12 19215723

[B11] E-alert 24 July Case of Lassa fever imported into Germany from Sierra Leone, 2006 Euro Surveill 2009 11 7 27 16966767

[B12] Macher AM Wolfe MS Historical Lassa fever reports and 30-year clinical update Emerg Infect Dis 2006 12 5 835 837 16704848 10.3201/eid1205.050052 PMC3374442

[B13] Ufberg JW Karras DJ Update on emerging infections: news from the Centers for Disease Control and Prevention. Imported Lassa fever--New Jersey, 2004 Ann Emerg Med 2005 45 3 323 326 10.1016/j.annemergmed.2004.12.015 15726058

[B14] Imported Lassa fever--New Jersey Centers for Disease Control and Prevention (CDC) MMWR Morb Mortal Wkly Rep 2004 53 38 894 897 15457145

[B15] Haas WH Breuer T Pfaff G Schmitz H Köhler P Asper M Emmerich P Drosten C Gölnitz U Fleischer K Günther S Imported Lassa fever in Germany: surveillance and management of contact persons Clin Infect Dis 2003 36 10 1254 1258 10.1086/374853 12746770

[B16] Hugonnet S Sax H Pittet D Management of viral haemorrhagic fevers in Switzerland Euro Surveill 2002 7 3 42 44 12631944 10.2807/esm.07.03.00340-en

[B17] Colebunders R Van Esbroeck M Moreau M Borchert M Imported viral haemorrhagic fever with a potential for person-to-person transmission: review and recommendations for initial management of a suspected case in Belgium Acta Clin Belg 2002 57 5 233 240 12534129 10.1179/acb.2002.047

[B18] Schmitz H Köhler B Laue T Drosten C Veldkamp PJ Günther S Emmerich P Geisen HP Fleischer K Beersma MF Hoerauf A Monitoring of clinical and laboratory data in two cases of imported Lassa fever Microbes Infect 2002 4 1 43 50 10.1016/S1286-4579(01)01508-8 11825774

[B19] Günther S Emmerich P Laue T Kühle O Asper M Jung A Grewing T ter Meulen J Schmitz H Imported lassa fever in Germany: molecular characterization of a new lassa virus strain Emerg Infect Dis 2000 6 5 466 476 10.3201/eid0605.000504 10998376 PMC2627947

[B20] Lassa fever, imported case, Netherlands Wkly Epidemiol Rec 2000 75 33 265 10974948

[B21] Lassa fever imported to England Commun Dis Rep CDR Wkly 2000 10 11 99 10769487

[B22] Lassa fever, case imported to Germany Wkly Epidemiol Rec 2000 75 3 17 18 10686828

[B23] Schmitz H Emmerich P ter Meulen J Imported tropical virus infections in Germany Arch Virol Suppl 1996 11 67 74 8800807 10.1007/978-3-7091-7482-1_8

[B24] Johnson KM Monath TP Imported Lassa fever-reexamining the algorithms N Engl J Med 1990 323 16 1139 1141 10.1056/NEJM199010183231611 2215584

[B25] Mahdy MS Chiang W McLaughlin B Derksen K Truxton BH Neg K Lassa fever: the first confirmed case imported into Canada Can Dis Wkly Rep 1989 15 39 193 198 2590947

[B26] Hirabayashi Y Oka S Goto H Shimada K Kurata T Fisher-Hoch SP McCormick JB The first imported case of Lassa fever in Japan Nippon Rinsho 1989 47 1 71 75 2724572

[B27] Hirabayashi Y Oka S Goto H Shimada K Kurata T Fisher-Hoch SP McCormick JB An imported case of Lassa fever with late appearance of polyserositis J Infect Dis 1988 158 4 872 875 10.1093/infdis/158.4.872 3171229

[B28] Zweighaft RM Fraser DW Hattwick MA Winkler WG Jordan WC Alter M Wolfe M Wulff H Johnson KM Lassa fever: response to an imported case N Engl J Med 1977 297 15 803 807 10.1056/NEJM197710132971504 895819

[B29] Bengtsson E Lassa fever-a new and contagious exotic imported disease Lakartidningen 1976 73 41 3425 3426 979408

[B30] Woodruff AW Monath TP Mahmoud AA Pain AK Morris CA Lassa fever in Britain: an imported case Br Med J 1973 3 5881 616 617 10.1136/bmj.3.5881.616 4755184 PMC1586880

[B31] Johnson KM McCormick JB Webb PA Smith ES Elliott LH King IJ Clinical virology of Lassa fever in hospitalized patients J Infect Dis 1987 155 3 456 464 10.1093/infdis/155.3.456 3805773

[B32] Shlaeffer F Sikuler E Keynan A Lassa fever--first case diagnosed in Israel Harefuah 1988 114 1 12 14 3350404

[B33] McCormick JB Webb PA Krebs JW Johnson KM Smith ES A prospective study of the epidemiology and ecology of Lassa fever J Infect Dis 1987 155 437 444 10.1093/infdis/155.3.437 3805771

[B34] McCormick JB Clinical, epidemiologic, and therapeutic aspects of Lassa fever Med Microbiol Immunol 1986 175 153 155 10.1007/BF02122438 3724661

[B35] Branco LM Matschiner A Fair JN Goba A Sampey DB Ferro PJ Cashman KA Schoepp RJ Tesh RB Bausch DG Garry RF Guttieri MC Bacterial-based systems for expression and purification of recombinant Lassa virus proteins of immunological relevance Virol J 2008 5 74 10.1186/1743-422X-5-74 18538016 PMC2435526

[B36] Illick MM Branco LM Fair JN Illick KA Matschiner A Schoepp R Garry RF Guttieri MC Uncoupling GP1 and GP2 expression in the Lassa virus glycoprotein complex: implications for GP1 ectodomain shedding Virol J 2008 5 161 10.1186/1743-422X-5-161 19105844 PMC2645378

[B37] Demby AH Chamberlain J Brown DW Clegg CS Early diagnosis of Lassa fever by reverse transcription-PCR J Clin Microbiol 1994 32 12 2898 2903 7883875 10.1128/jcm.32.12.2898-2903.1994 PMC264198

[B38] Trappier SG Conaty AL Farrar BB Auperin DD McCormick JB Fisher-Hoch SP Evaluation of the polymerase chain reaction for diagnosis of Lassa virus infection Am J Trop Med Hyg 1993 49 2 214 221 8357084 10.4269/ajtmh.1993.49.214

[B39] Edgar RC MUSCLE: a multiple sequence alignment method with reduced time and space complexity BMC Bioinformatics 2004 19 5 113 10.1186/1471-2105-5-113 PMC517706 15318951

[B40] Grove JN Boisen ML Muncy IJ Henderson LA Schiefellin JS Robinson JE Bangura JJ Fonnie M Schoepp RJ Hensley LE Seisay A Fair JN Garry RF Capacity building permitting comprehensive monitoring of a severe case of Lassa hemorrhagic fever in Sierra Leone with a positive outcome: case report Virol J 2011 8 314 10.1186/1743-422X-8-314 21689444 PMC3283910

[B41] Ter Meulen J Koulemou K Wittekindt T Windisch K Strigl S Conde S Schmitz HJ Detection of Lassa virus antinucleoprotein immunoglobulin G (IgG) and IgM antibodies by a simple recombinant immunoblot assay for field use Clin Microbiol 2001 36 11 3143 3148 10.1128/jcm.36.11.3143-3148.1998 PMC105290 9774554

[B42] Günther S Kühle O Rehder D Odaibo GN Olaleye DO Emmerich P ter Meulen J Schmitz H Antibodies to Lassa virus Z protein and nucleoprotein co-occur in human sera from Lassa fever endemic regions Med Microbiol Immunol 2001 189 4 225 229 10.1007/s004300100061 11599793

[B43] Jahrling PB Evans AS, Kaslow RA Acute viral infections: Arenaviruses Virus infections of humans: epidemiology and control 1997 4 New York: Plenum 199 209

[B44] Mahanty S Bausch DG Thomas RL Goba A Bah A Peters CJ Rollin PE Low levels of Interleukin-8 and Interferon-inducible protein-10 in serum are associated with fatal infections in acute Lassa fever J Inf Dis 2001 183 1713 1721 10.1086/320722 11372023

[B45] Walker D Jason J Wallace K Slaughter J Whatley V Han A Nwanyanwu OC Kazembe PN Dobbie H Archibald L Jarvis WR Spontaneous Cytokine Production and Its Effect on Induced Production Clin Diag Lab Immunol 2002 9 5 1049 1056 10.1128/CDLI.9.5.1049-1056.2002 PMC120078 12204958

[B46] Slowik MR Min W Ardito T Karsan A Kashgarian M Pober JS Evidence that tumor necrosis factor triggers apoptosis in human endothelial cells by interleukin-1-converting enzyme-like protease-dependent and -independent pathways Lab Invest 1997 77 257 267 9314949

[B47] Michelmann I Böckmann D Nürnberger W Eckhof-Donovan S Burdach S Göbel U Thrombocytopenia and complement activation under recombinant TNF alpha/IFN gamma therapy in man Ann Hematol 1997 74 179 184 10.1007/s002770050279 9174546

[B48] Price ME Fisher-Hoch SP Craven RB McCormick JB A prospective study of maternal and fetal outcome in acute Lassa fever infection during pregnancy BMJ 1988 297 6648 584 587 10.1136/bmj.297.6648.584 3139220 PMC1834487

