# Correction: Lassa hemorrhagic fever in a late term pregnancy from northern Sierra Leone with a positive maternal outcome: case report

**DOI:** 10.1186/1743-422X-8-480

**Published:** 2011-10-25

**Authors:** Luis M Branco, Matt L Boisen, Kristian G Andersen, Jessica N Grove, Lina M Moses, Ivana J Muncy, Lee A Henderson, John S Schieffellin, James E Robinson, James J Bangura, Donald S Grant, Vanessa N Raabe, balu M Fonnie, Eleina M Zaitsev, Pardis C Sabeti, Robert F Garry

**Affiliations:** 1 Department of Microbiology and Immunology, Tulane University, New Orleans, Louisiana, USA; 2 Autoimmune Technologies, LLC, New Orleans, Louisiana, USA; 3 Corgenix Medical Corporation, Broomfield, Colorado, USA; 4 Department of Organismic and Evolutionary Biology, Center for Systems Biology, Harvard University, Cambridge, Massachusetts, USA; 5 Vybion, Inc., Ithaca, New York, USA; 6 Department of Paediatrics, Section of Infectious Disease, Tulane University, New Orleans, Louisiana, USA; 7 Ministry of Health and Sanitation Workplace Health, Republic of Sierra Leone, Freetown, Sierra Leone; 8 The Global Viral Forecasting Initiative, San Francisco, California, USA; 9 Kenema Government Hospital Lassa Fever Ward, Kenema, Republic of Sierra Leone; 10 University of Minnesota School of Medicine, Minneapolis, Minnesota, USA; 11 Broad Institute of Massachusetts Institute of Technology and Harvard, Cambridge, Massachusetts, USA

## Correction

After publication of this work [1], we noted that we inadvertently failed to include the complete list of all coauthors. The full list of authors has now been added and the Authors' contributions and Competing interests section modified accordingly.

## Competing interests

The authors declare that they have no competing interests.

## Authors' contributions

Conceived and designed the experiments: LMB, MLB, KGA, RFG. Performed the experiments: LMB, MLB, KGA, EMZ. Analyzed the data/critical review of manuscript: LMB, MLB, KGA, JNG, JSS, JER, DSG, VNR, PCS, RFG. Contributed reagents/materials: IJM, LAH. Provided medical/outreach/case investigation support in Sierra Leone: LMM, JJB, DSG, VNR, MF. Wrote the manuscript: LMB, MLB, KGA, JNG, RFG. All authors have read and approved the final manuscript.
