# Supplementary material for: Lassa hemorrhagic fever in a late term pregnancy from northern sierra leone with a positive maternal outcome: case report
Source: Virol J. 2011 Aug 15;8:404. doi: 10.1186/1743-422X-8-404 (PMC3177908; doi:10.1186/1743-422X-8-404)
Supplement: Additional File 1 — Map of Sierra Leone and expanded view of relevant localities and routes travelled by patient G-1442. Maps of Sierra Leone outlining Districts (A) and Provinces (B) [http://commons.wikimedia.org/wiki/Atlas_of_Sierra_Leone], with an inset map (C) [http://maps.google.com] displaying the location of Mabineh 1 [red star], where the suspected LF case in the current report originated, and the four localities where the patient travelled to and from, with known dates noted: Waterloo (late Dec 2010), Masingbi (early Jan 2011), Tongo (Jan 10, 2011), and Kenema (Jan 19, 2011). The inbound routes travelled by the patient are indicated in dotted lines, and outbound ones in solid lines. The bar represents 20 miles. [file 1743-422X-8-404-S1.PPT]

## Slide 1
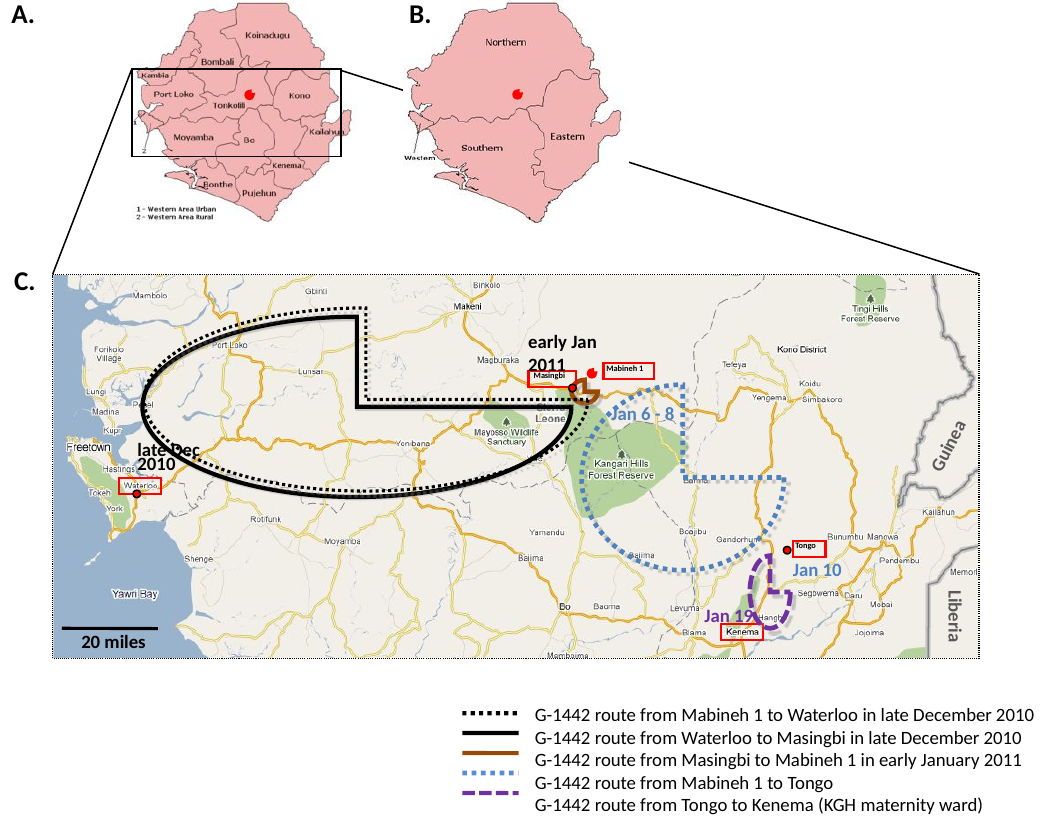

A.
B.
C.
early Jan
2011
Mabineh 1
Masingbi
Jan 6 - 8
Guinea
late Dec
2010
Tongo
Jan 10
Jan 19
Liberia
20 miles
G-1442 route from Mabineh 1 to Waterloo in late December 2010
G-1442 route from Waterloo to Masingbi in late December 2010
G-1442 route from Masingbi to Mabineh 1 in early January 2011
G-1442 route from Mabineh 1 to Tongo
G-1442 route from Tongo to Kenema (KGH maternity ward)
