# Supplementary figures and images for: Lassa hemorrhagic fever in a late term pregnancy from northern sierra leone with a positive maternal outcome: case report
Source: Virol J. 2011 Aug 15;8:404. doi: 10.1186/1743-422X-8-404 (PMC3177908; doi:10.1186/1743-422X-8-404)

## Slide 1
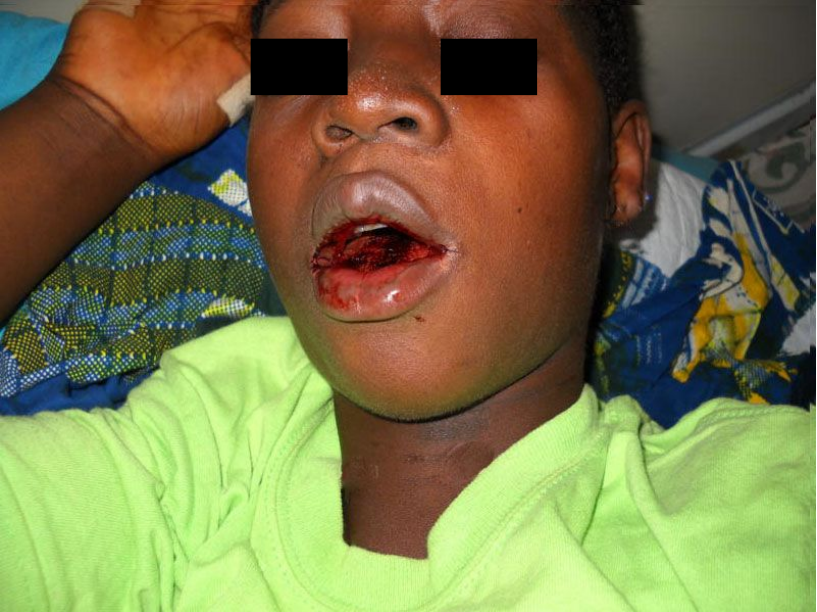

Supplement: Additional File 2 — Patient G-1442 at time of admission presenting with haemoptysis, facial edema, gingivorrhagia. Patient G-1442 presented with significant haemoptysis, facial edema, and gingivorrhagia, at the time of admission and medical assessment at the KGH LFW. These symptoms persisted for several days after admission but resolved with ribavirin treatment. [file 1743-422X-8-404-S2.PPT]

## Slide 1
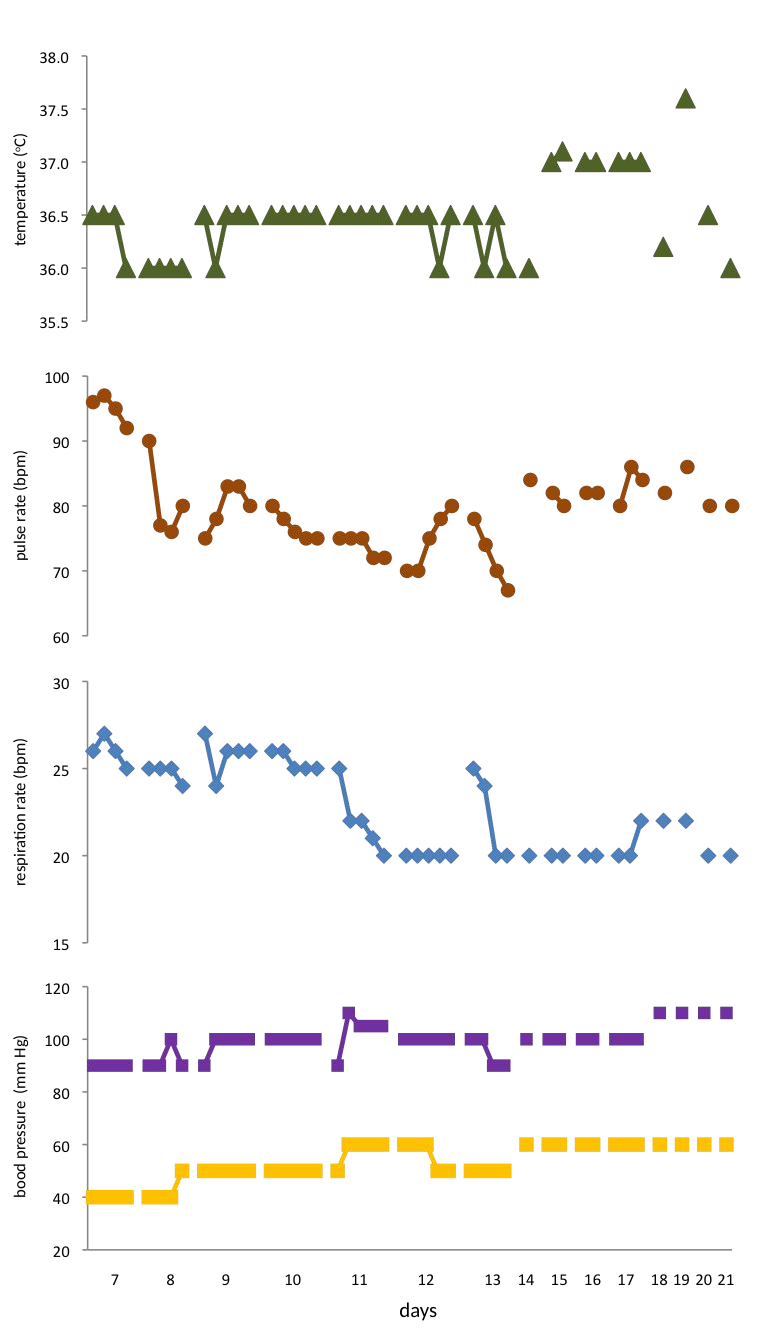

temperature (oC)
pulse rate (bpm)
respiration rate (bpm)
bood pressure (mm Hg)
days

Supplement: Additional Figure 3 — Vital signs for G-1442 during hospitalization at KGH LFW. Core temperature (°C) [green triangle], pulse [red circle], respiratory rate [blue diamond], and blood pressure purple [square = systolic, yellow square = diastolic] were measured at regular intervals, usually every 4 hours at the onset, and every 12 hours at later times, throughout the hospitalization period. [file 1743-422X-8-404-S3.PPT]

## Slide 1
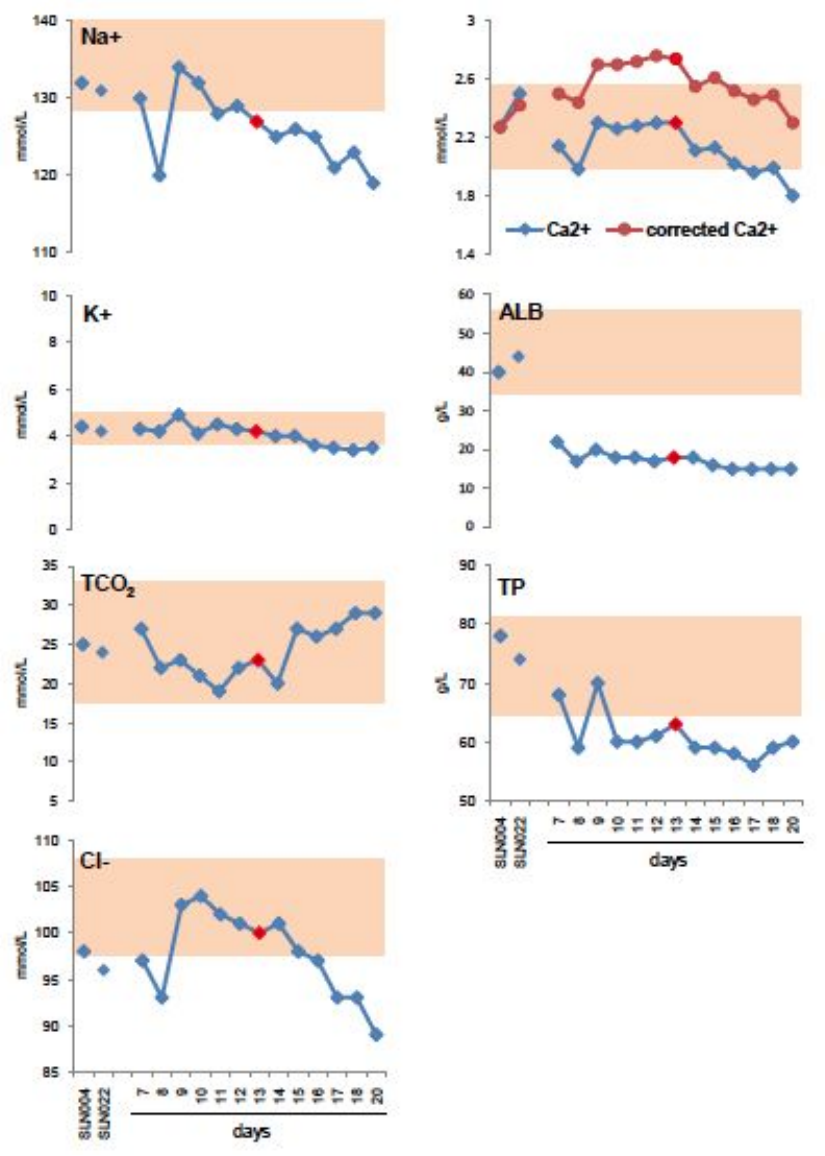

Supplement: Additional Figure 4 — Additional Piccolo metabolites analyzed in G-1442. Patient G-1442 presented with low serum Cl- and albumin, normal K+, Na+, Ca2+ (corrected for albumin levels), TCO2, and total protein levels. Over the course of disease management the patient developed hyponatremia, hypochloremia, and slight hypokalemia. Total protein and albumin levels remained low throughout. Between days 9 and 13 G-1442 developed hypercalcaemia, but then normalized. Metabolic indicators in the two healthy Sierra Leonean donors all were within or near normal ranges. [file 1743-422X-8-404-S4.PPT]

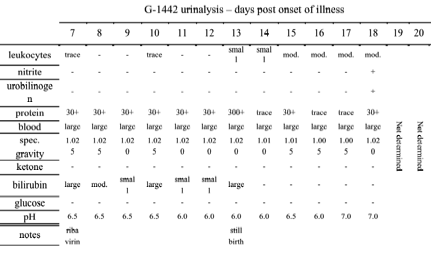

Supplement: Additional Figure 5 — Table 1. Urinalysis profile for patient G-1442 during the course of admission at the KGH LFW. Urine samples were collected from patient G-1442 daily (days 7-18) and tested for 10 metabolites as outlined in Methods. The first day of ribavirin administration (7) and still birth delivery (13) are noted. Abbreviations and codes: moderate (mod.); negative (-); positive (+); specific gravity (spec. gravity); 30 mg/dL protein in urine (30+); 300 mg/dL protein in urine (300+). [file 1743-422X-8-404-S5.DOCX]
